# Global burden of cardiovascular disease mortality attributable to secondhand smoke, 1990–2019: Systematic analysis of the Global Burden of Disease Study 2019

**DOI:** 10.1371/journal.pone.0316023

**Published:** 2024-12-27

**Authors:** Juan Dong, Xumin Ma, Xingxin Hu, Mengmeng Yan

**Affiliations:** 1 Southwest Medical University, Luzhou, China; 2 Meishan Cancer Hospital, Meishan, China; 3 Urban Vocational College of Sichuan, Chengdu, China; 4 Chengdu University of TCM, Chengdu, China; 5 University of Electronic Science and Technology of China, Chengdu, China; National center for chronic and non-communicable diesease prevention and control, CHINA

## Abstract

**Background:**

Few studies have globally assessed the cardiovascular disease (CVD) mortality burden attributable to secondhand smoke. We aimed to address this research gap.

**Methods:**

We used a systematic analysis design using data from the Global Burden of Disease Study 2019. Our primary outcome measures were the age-standardized mortality rate (ASMR) and age-standardized disability-adjusted life years (DALYs) for CVD attributable to secondhand smoke. The annual average percentage change (AAPC) was utilized to describe the temporal trends of ASMR and DALYs.

**Results:**

From 1990 to 2019, global ASMR for CVD due to secondhand smoke decreased from 11.45 (95% CI: 9.47 to 13.42) to 7.43 (95% CI: 6.09 to 8.85), and DALYs decreased from 274.12 (95% CI: 225.36 to 322.20) to 176.93 (95% CI: 145.21 to 211.28). ASMR and DALYs attributable to secondhand smoke are on the rise in 47 countries, with 18 of these countries experiencing increases across both genders and all cardiovascular subtypes. Uzbekistan, Lesotho, and the Philippines have the highest AAPC for CVD due to secondhand smoke in ASMR and DALYs. Specifically, Uzbekistan’s overall ASMR AAPC is 2.2 (95%CI: 2.1–2.3), Lesotho’s is 1.3 (95%CI: 1.2–1.3), and the Philippines’ is 1.1 (95%CI: 1.0–1.2). In terms of DALYs, the AAPC values are 1.7 for Uzbekistan (95%CI: 1.7–1.8), 1.4 for Lesotho (95%CI: 1.3–1.5), and 1.8 for the Philippines (95%CI: 1.7–1.9).

**Conclusion:**

Over the past three decades, the epidemiological landscape of CVD mortality associated with secondhand smoke has undergone significant shifts. Notwithstanding global advancements, intensified interventions are paramount in regions experiencing ascending rates.

## Introduction

Cardiovascular diseases (CVD) are the leading cause of death globally, posing a significant impact on global public health [1]. According to the World Health Organization, CVD account for approximately 17.9 million deaths each year, representing 31% of all global deaths [2]. These diseases’ incidence and mortality rates are influenced by various risk factors, including smoking, hypertension, hyperlipidemia, diabetes, obesity, and unhealthy dietary habits [3]. Although there has been extensive research on the impact of smoking on CVD, the effect of secondhand smoke on CVD mortality has not been adequately addressed [4].

Secondhand smoke, also known as environmental tobacco smoke, refers to the tobacco smoke inhaled by non-smokers, including mainstream smoke (smoke exhaled by smokers) and sidestream smoke (smoke from burning tobacco products) [5]. It is estimated that a quarter of the global adult population and nearly half of all children are exposed to secondhand smoke [6]. Secondhand smoke has been proven to be associated with various health problems, including CVD, lung cancer, and respiratory diseases [7].

The impact of secondhand smoke on CVD has been extensively studied, and evidence suggests that secondhand smoke can increase the risk of CVD [8]. However, these studies are often limited to specific regions or populations, and there is a paucity of global studies, particularly regarding the impact of secondhand smoke on CVD mortality [9, 10].

Therefore, using data from the Global Burden of Disease Study 2019, we conducted a systematic analysis of secondhand smoke on the burden of CVD mortality from 1990 to 2019.

## Methods

### Study design and data source

This study is a systematic analysis of the Global Burden of Disease Study (GBD) 2019 data. The GBD 2019 is a comprehensive regional and global research program of disease burden that assesses mortality and disability from major diseases, injuries, and risk factors [11].

### Definition of secondhand smoke exposure

Secondhand smoke exposure is defined as current exposure to secondhand tobacco smoke at home, at work, or in other public places [12]. Non-smokers, including ex-smokers and occasional smokers, living with a daily smoker or working in a place where smoking is allowed are considered exposed to secondhand smoke. Both children and adults are included in the exposure assessment.

### Estimation of CVD burden attributable to secondhand smoke

CVD encompasses a range of ICD (International Classification of Diseases, a WHO coding system that classifies diseases and health conditions, assigning each a unique code for consistency in medical records, public health statistics, and monitoring) codes, including B33.2, G45-G46.8, I01-I01.9, I02.0, I05-I09.9, I11-I11.9, I20-I25.9, I27.0, I27.2, I28-I28.9, I30-I31.1, I31.8-I37.8, I38-I41.9, I42.1-I42.8, I43-I43.9, I47-I48.9, I51.0-I51.4, I60-I63.9, I65-I66.9, I67.0-I67.3, I67.5-I67.6, I68.0-I68.2, I69.0-I69.3, I70.2-I70.8, I71-I73.9, I77-I83.9, I86-I89.0, I89.9, I98, and K75.1. Specifically, ischemic heart disease is represented by ICD codes I20-I25.9, while stroke is represented by G45-G46.8, I60-I63.9, I65-I66.9, I67.0-I67.3, I67.5-I67.6, I68.1-I68.2, and I69.0-I69.3.

The burden of CVD attributable to secondhand smoke is estimated using a systematic approach [12]. The proportion of non-smokers living with at least one smoker is calculated using data on household composition from representative major survey series and national and subnational censuses. The proportion of individuals exposed to secondhand smoke at work is estimated using self-reported data from cross-sectional surveys. The probability of an individual being exposed to secondhand smoke, either through non-occupational or occupational exposure, is calculated. This probability is then multiplied by the probability that the individual is not a smoker, resulting in individual-level probabilities of exposure. These probabilities are then averaged to produce probabilities of exposure by location, year, age, and sex. The burden of CVD attributable to secondhand smoke is then estimated using the standard GBD population attributable fraction (PAF) equation, which takes into account both the exposure probabilities and relative risks of CVD for exposed versus non-exposed individuals. The theoretical minimum-risk exposure level for secondhand smoke is considered to be zero exposure among non-smokers.

### Outcome measures

The primary outcome measures are the age-standardized mortality rate (ASMR) and age-standardized disability-adjusted life years (DALYs) attributable to secondhand smoke. These measures are stratified by different CVD types and gender. The focus of CVD in this study primarily includes ischemic heart disease and stroke, as these are among the most common and impactful cardiovascular conditions globally.

### Statistical analysis

We utilized the ASMR and age-standardized DALYs provided by the GBD 2019. These rates have been standardized based on the world standard population reported in the study and are presented per 100,000 population. Temporal trends in the ASMR and DALYs for CVD attributable to secondhand smoke were assessed using joinpoint regression. The Joinpoint Regression Program software was used to fit the simplest joinpoint model that the data allowed. The program started with the minimum number of joinpoints (e.g., a straight line of 0 joinpoint) and tested whether more joinpoints were statistically significant and should be added to the model. A Monte Carlo permutation method was used for tests of significance. The average annual percent change was calculated from the annual percent change of each segment in the last significant model in a weighted manner.

All statistical analyses were performed using R software (version 4.1.0) and Joinpoint Regression Program software (version 4.9.1.0, National Cancer Institute, USA), with a P value <0.05 considered statistically significant.

## Results

### Global distribution of CVD attributable to secondhand smoke in 2019

**Fig 1:**
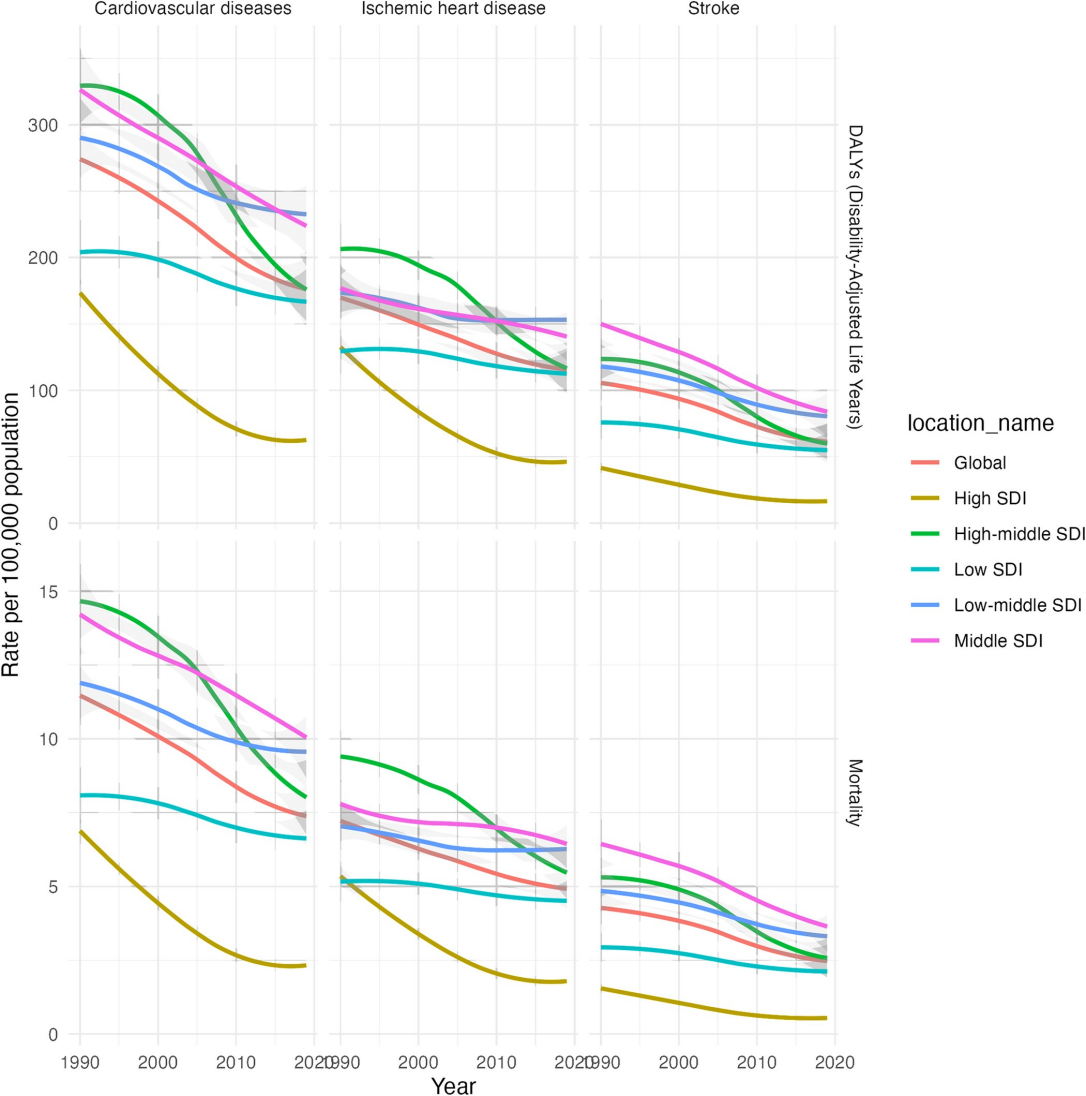
ASMR and age-standardized DALYs for cardiovascular diseases attributable to secondhand smoke, 1990–2019.

In 2019, the global ASMR attributable to CVD due to secondhand smoke was 7.43 (95% CI 6.09 to 8.85) per 100,000 population (Fig 1). The mortality rate for ischemic heart disease was 4.94 (95% CI 3.98 to 5.93), and for stroke, it was 2.49 (95% CI 1.86 to 3.18). The same year (S1, S2 Figs), the global age-standardized DALYs for CVD due to secondhand smoke was 176.93 (95% CI 145.21 to 211.28) per 100,000 population, with ischemic heart disease contributing 115.48 (95% CI 93.33 to 139.01) and stroke contributing 61.46 (95% CI 45.97 to 78.78).

Within the SDI categories (Fig 1), the low-middle SDI category had the highest ASMR for CVD due to secondhand smoke, at 9.51 (95% CI 7.71 to 11.47) per 100,000 population, and the DALYs was 230.63 (95% CI 186.10 to 278.72). Conversely, the high SDI category had the lowest ASMR for CVD due to secondhand smoke, at 2.33 (95% CI 1.93 to 2.71) per 100,000 population, and the DALYs was 62.38 (95% CI 51.85 to 73.15).

When considering specific types of CVD, the ASMR (Fig 1) and DALYs for ischemic heart disease were higher than for stroke across all SDI categories. For instance, in the low-middle SDI category, the ASMR for ischemic heart disease was 6.20 (95% CI 4.94 to 7.53) per 100,000 population, and the DALYs was 151.13 (95% CI 120.18 to 183.83). Correspondingly, the ASMR for stroke was 3.30 (95% CI 2.41 to 4.25) per 100,000 population, and the DALYs was 79.51 (95% CI 58.86 to 104.10).

At the regional level, the greatest death burden from secondhand smoke-induced CVD and ischemic heart disease was observed in parts of Oceania and Central Asia (S1 Table).

At the national level, the eight countries with the highest ASMR, and age-standardized DALYs in 2019 were predominantly from Oceania and Central Asia (e.g., Solomon Islands, Kiribati, Nauru, and Turkmenistan), making these regions bear the greatest burden of deaths from secondhand smoke-induced CVD and ischemic heart disease (S2 Table). In 2019, these countries experienced the highest ASMR attributed to secondhand smoke-induced CVD: Solomon Islands (41.20; 95% CI: 31.49–52.79), Kiribati (31.55; 95% CI: 23.31–40.70), Nauru (30.15; 95% CI: 23.13–38.81), Turkmenistan (30.05; 95% CI: 23.26–38.14), Azerbaijan (29.55; 95% CI: 23.57–36.22), Uzbekistan (28.89; 95% CI: 22.77–35.66), Afghanistan (24.53; 95% CI: 18.17–31.40), Yemen (22.98; 95% CI: 17.49–30.28), Micronesia (Federated States of) (22.81; 95% CI: 15.85–31.33), and Tuvalu (22.50; 95% CI: 16.51–29.66). Additionally, these countries showed the highest DALY attributed to the same cause. Gender disparities in ASMR attributed to secondhand smoke-induced CVD were observed among the top 10 countries in 2019. For instance, in the Solomon Islands, the death rate was 41.57 (95% CI: 30.99–54.50) for males and 40.80 (95% CI: 30.11–52.19) for females. Similarly, in Kiribati, the rates were 37.48 (95% CI: 27.89–48.67) for males and 26.40 (95% CI: 19.23–34.72) for females. Turkmenistan, Nauru, and other countries also exhibited notable gender disparities in these death rates.

### Temporal trend of CVD attributable to secondhand smoke from 1990 to 2019

Globally, from 1990 to 2019, there was a significant decline in ASMR and DALYs attributed to secondhand smoke for CVD (Fig 1). The ASMR decreased from 11.45 (95% CI: 9.47 to 13.42) to 7.43 (95% CI: 6.09 to 8.85), and the age-standardized DALYs rate decreased from 274.12 (95% CI: 225.36 to 322.20) to 176.93 (95% CI: 145.21 to 211.28). Similar trends were observed for ischemic heart disease and stroke. In high SDI regions, regardless of gender or disease type, the AAPC for ASMR are notably substantial, such as an AAPC of -3.7% (95% CI: -3.7 to -3.6) for CVD. However, as the SDI level decreases, the AAPC values gradually diminish, with CVD showing an AAPC of -0.7% (95% CI: -0.7 to -0.7) in low SDI areas. The AAPC for DALYs appear to follow a similar pattern to those observed for ASMR changes.

Multiple regions are distinctly displaying an upward trajectory in ASMR, as indicated by their AAPC. Notably, regions like East Asia, Oceania, Central Asia, and Southeast Asia showcase this trend (S3 Table). Specifically, the AAPC for male ischemic heart disease are 0.4 (East Asia, 95% CI: 0.3 to 0.4), 0.2 (Central Asia, 95% CI: 0.1 to 0.3), and 0.1 (Southeast Asia, 95% CI: 0.1 to 0.1). Furthermore, both the overall and female ischemic heart disease AAPC values in the Oceania region stand at 0.3 (95% CI: 0.3 to 0.3). Shifting to AAPC for DALYs, intriguing patterns emerge. For instance, ischemic heart disease DALYs for females in Oceania show a 0.4 increase (95% CI: 0.4 to 0.4). In Eastern Europe, there is a 0.3 rise in ischemic heart disease DALYs for males (95% CI: 0.0 to 0.5). Similarly, cardiovascular disease DALYs in Oceania witness increments of 0.2 (95% CI: 0.1 to 0.2) for females and 0.1 (95% CI: 0.0 to 0.1) for males. Notably, Southeast Asia demonstrates a significant surge in male ischemic heart disease DALYs, reflecting an increase of 0.2 (95% CI: 0.1 to 0.2).

**Fig 2:**
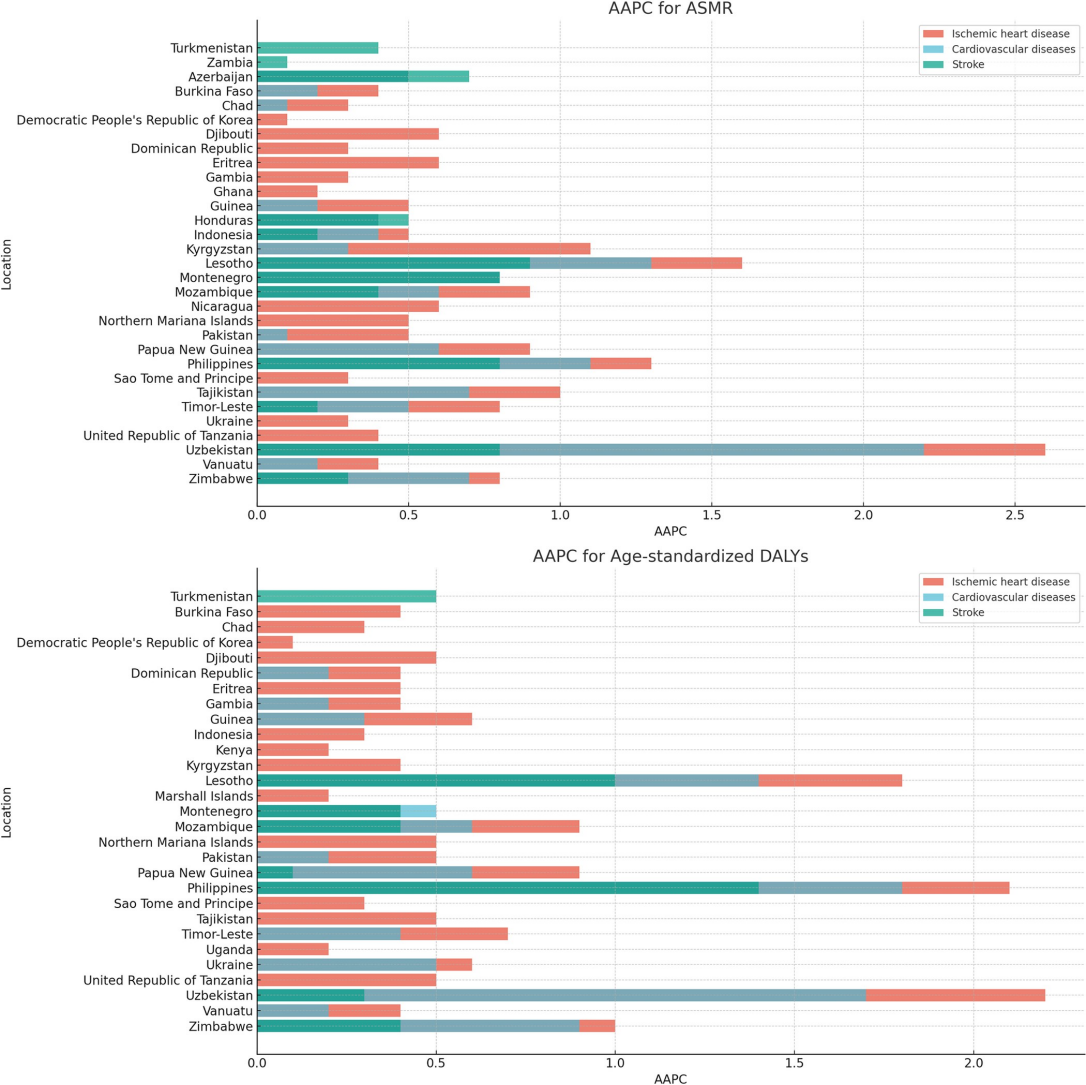
AAPC of ASMR and age-standardized DALYs for cardiovascular diseases attributable to secondhand smoke in countries with increasing trends.

In terms of specific countries, there is a growing trend of increased attributable secondhand smoke-related ASMR and DALYs in 47 countries (Fig 2). Among these, 18 countries show an increase in ASMR for both genders and cardiovascular disease subtypes (S4 Table and S3, S4 Figs). Furthermore, the number of countries with an increased burden of ischemic heart disease attributable to secondhand smoke exceeds that of stroke (Fig 2).

Uzbekistan, Lesotho, and the Philippines are the top three countries with the highest AAPC values for CVD attributed to second-hand smoke, both in terms of ASMR and DALYs (Fig 2). For ASMR (S4 Table and S3 Fig): In Uzbekistan, the overall AAPC for CVD among both sexes is 2.2 (95%CI: 2.1–2.3). When comparing by gender, men have a slightly higher AAPC for CVD at 2.3 (95%CI: 2.1–2.4) compared to women’s 2.2 (95%CI: 2.1–2.3). For ischemic heart disease and stroke, the differences between the genders are not significant. In Lesotho, the overall AAPC for CVD among both sexes is 1.3 (95%CI: 1.2–1.3). Comparatively, women have significantly higher AAPC values than men, especially for ischemic heart disease where women’s AAPC is 2.1 (95%CI: 2.0–2.2) compared to men’s 1.0 (95%CI: 1.0–1.1). In the Philippines, the overall AAPC for CVD among both sexes is 1.1 (95%CI: 1.0–1.2). Contrarily, men consistently have slightly higher AAPC values across all indicators compared to women. For DALYs (S4 Table and S4 Fig): In Uzbekistan, the overall AAPC for CVD among both sexes is 1.7 (95%CI: 1.7–1.8). Men have a slightly higher AAPC for CVD and stroke compared to women, especially in stroke where men’s AAPC is 0.8 (95%CI: 0.7–0.9) compared to women’s 0.0 (95%CI: -0.1–0.1). In Lesotho, the overall AAPC for CVD among both sexes is 1.4 (95%CI: 1.3–1.5). Similar to the trend in ASMR, women have significantly higher AAPC values across all three indicators compared to men. In the Philippines, the overall AAPC for CVD among both sexes is 1.8 (95%CI: 1.7–1.9). Men consistently have higher AAPC values across all indicators compared to women.

By cardiovascular disease subtype, the AAPC of DALYs and ASMR attributable to secondhand smoke for ischemic heart disease and stroke showed a declining trend in most regions from 1990 to 2019 (S3 Table). However, an increasing trend in the AAPC for ischemic heart disease was observed in Oceania, with DALYs and ASMR rising at rates of 0.3 (95%CI: 0.2–0.3) and 0.1 (95%CI: 0.0–0.1), respectively. At the national level, the burden of ischemic heart disease attributable to secondhand smoke increased significantly in Uzbekistan, the Philippines, and Lesotho. The DALYs for these countries were 2.2 (95%CI: 2.1–2.3) in Uzbekistan, 2.1 (95%CI: 2.0, 2.2) in the Philippines, and 1.8 (95%CI: 1.8–1.9) in Lesotho. Similarly, the ASMR were 2.6 (95%CI: 2.4–2.7) in Uzbekistan, 1.3 (95%CI: 1.2–1.3) in the Philippines, and 1.6 (95%CI: 1.6–1.7) in Lesotho. For stroke, notable increases in the AAPC of DALYs were observed in the Philippines and Lesotho, with values of 1.4 (95%CI: 1.3–1.5) and 1.0 (95%CI: 0.9–1.0), respectively. The ASMR in these two countries were 0.8 (95%CI: 0.7–0.9) for the Philippines and 0.9 (95%CI: 0.9–1.0) for Lesotho.

## Discussion

### Key results and study objectives

Our study aimed to provide a comprehensive analysis of CVD mortality burden attributable to secondhand smoke exposure from 1990 to 2019. This three-decade span offered a unique vantage point to observe shifts and trends in the global health landscape. The key findings of our study are multifaceted:

Global Decline: On a global scale, there was a discernible decline in CVD mortality due to secondhand smoke over the thirty-year period. The ASMR diminished from 11.45 to 7.43 per 100,000 population, a significant drop that underscores the progress made in public health campaigns and initiatives targeting tobacco use and exposure [13].

Regional Disparities: Contrasting this global trend, there were alarming spikes in mortality rates in specific regions, most notably in parts of Oceania and Central Asia. Countries like Uzbekistan, Lesotho, and the Philippines exhibited increasing trends, highlighting the region-specific challenges and the need for tailored interventions.

Gender Disparities: Furthermore, our findings elucidated notable gender disparities, especially in high-risk countries. For instance, in the Solomon Islands and Kiribati, male mortality rates were significantly higher than their female counterparts. These gender-specific trends might be influenced by socio-cultural factors, behavioral patterns, or other underlying determinants.

Temporal Trends: Our temporal analysis underscored the dynamic nature of CVD mortality trends. While the overall decline is commendable, the uptick in specific regions during the recent years of the study period indicates evolving challenges, possibly due to changing socio-economic factors, healthcare access, or shifts in tobacco industry strategies.

### Mechanism of secondhand smoke leading to cardiovascular disease

Recent studies have corroborated that exposure to secondhand smoke is closely linked to the onset and progression of CVD. Harmful chemicals present in secondhand smoke, including nicotine, carbon monoxide, and oxygen-free radicals, have been proven detrimental to cardiac and vascular health [14]. These substances can cause vasoconstriction, elevated blood pressure, cardiac arrhythmias, and thrombogenesis, consequently escalating the risk of cardiovascular events [15]. Additionally, secondhand smoke may promote atherosclerosis by enhancing the oxidation of low-density lipoproteins and instigating inflammatory processes [16].

The observed increase in secondhand smoke-related mortality in certain regions can be attributed to a combination of socio-economic, cultural, and environmental factors. In some areas, tobacco smuggling has led to the widespread availability of harmful substances, while inadequate public awareness of the dangers of secondhand smoke further exacerbates exposure risks. Poor living conditions and limited healthcare resources also play significant roles, as these regions often struggle to provide adequate healthcare or preventive measures. In addition, high smoking rates, coupled with weak enforcement of smoking bans, contribute to the increased exposure, especially in densely populated urban areas. These challenges are compounded by local cultural and social habits that may tolerate smoking, as well as influences from neighboring countries with high smoking rates. Together, these factors increase secondhand smoke exposure and the associated health risks, necessitating targeted interventions that address these specific regional issues.

The decline in the CVD burden attributable to secondhand smoke in some regions can be attributed to several key factors. Stringent public health policies, such as smoking bans in public places and improved air quality standards, have played a significant role in reducing exposure to secondhand smoke. Furthermore, heightened awareness of the health risks associated with smoking and secondhand smoke has led to shifts in behavior and attitudes, contributing to a reduction in smoking prevalence and, consequently, a decrease in CVD-related mortality in certain areas.

The gender-specific trends may be influenced by social, cultural, and behavioral factors. In these countries, men are often exposed to higher levels of secondhand smoke, which could be attributed to cultural norms around smoking behavior. Additionally, the marketing strategies of the tobacco industry may exacerbate these trends [17]. While some countries have taken steps to regulate the tobacco industry, in others, the industry continues to influence public policy and promotes smoking through targeted campaigns, which may further increase smoking prevalence among men. These combined factors likely contribute to the gender disparities observed in the mortality rates related to secondhand smoke exposure.

### Study limitations

Our research, primarily based on observational data, does not establish causality. Although extensive data was utilized, potential biases may have arisen due to data incompleteness and inaccuracies. Moreover, unconsidered confounding factors in our analysis might have influenced our results [18]. Specifically, one limitation of our study is the lack of granular regional data, which prevents us from identifying local factors contributing to the rise in CVD mortality from secondhand smoke. Additionally, the GBD data used does not include information on cardiovascular disease subtypes beyond ischemic heart disease and stroke.

### Interpretation and insights

Interpreting our findings, it’s paramount to recognize the intricacies of global CVD. While the overall trend suggests a decline in mortality, the escalating trend in certain countries and regions remains a pressing public health concern [19]. This implies that, even though global tobacco control strategies might be showing efficacy, more rigorous efforts and resources are needed in specific nations and territories to counteract the burden of CVD induced by secondhand smoke [20].

### Comparison with relevant studies

Consistent with other contemporary studies, our findings report a global decline in cardiovascular disease mortality attributed to secondhand smoke [21]. However, discrepancies were identified when comparing our results with studies focusing on specific countries or regions [22, 23]. These variations could be attributed to differences in research design, data sources, or statistical methodologies [24].

### Generalizability of results

While our research findings have global implications, their generalizability may be limited in specific cultural, economic, and health contexts. Distinct health behaviors, tobacco use habits, and cardiovascular health risks in certain communities or populations might affect the application and interpretation of our study outcomes [25, 26].

### Conclusion

Our research underscores the evolving landscape of cardiovascular disease mortality attributable to secondhand smoke over the past three decades. While the global community has made commendable progress, specific regions continue to grapple with increasing rates. Effective, targeted interventions, especially in these high-burden areas, coupled with sustained global efforts, are imperative to mitigate this public health menace.

## Supporting information

S1 Fig DALYs by location, 2019. (DOCX)

S2 Fig ASMR by location, 2019. (DOCX)

S3 Fig AAPC of ASMR for cardiovascular diseases attributable to secondhand smoke, stratified by gender and cardiovascular disease type. (DOCX)

S4 Fig AAPC of age-standardized DALYs for cardiovascular diseases attributable to secondhand smoke, stratified by gender and cardiovascular disease type. (DOCX)

S1 Table Burden of cardiovascular diseases attributable to secondhand smoke by region, in 2019. (DOCX)

S2 Table Countries with an increased burden of cardiovascular disease attributable to second-hand smoke, by gender. (DOCX)

S3 Table AAPC of age-standardized cardiovascular disease mortality and DALYs attributable to secondhand smoke, stratified by region, 1990–2019. (DOCX)

S4 Table Countries with an increased burden of cardiovascular disease deaths attributable to second-hand smoke, by type of cardiovascular disease and gender. (DOCX)
