# Correction: Global burden of cardiovascular disease mortality attributable to secondhand smoke, 1990–2019: Systematic analysis of the Global Burden of Disease Study 2019

**DOI:** 10.1371/journal.pone.0344100

**Published:** 2026-02-27

**Authors:** Juan Dong, Xumin Ma, Xingxin Hu, Mengmeng Yan

The first two authors, Juan Dong and Xumin Ma, should not have been attributed co-first authors to this work.
