# Supplementary figures and images for: Global burden of cardiovascular disease mortality attributable to secondhand smoke, 1990–2019: Systematic analysis of the Global Burden of Disease Study 2019
Source: PLoS One. 2024 Dec 27;19(12):e0316023. doi: 10.1371/journal.pone.0316023 (PMC11676574; doi:10.1371/journal.pone.0316023)

S1 Fig. DALYs by location, 2019


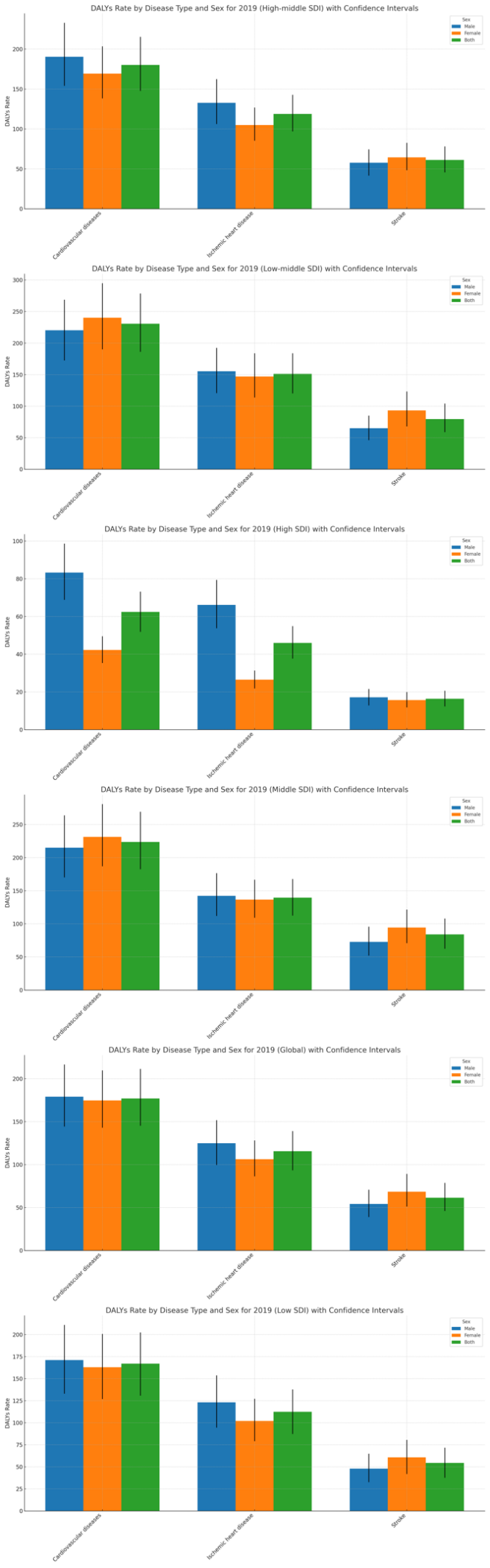

Supplement: S1 Fig — (DOCX) [file pone.0316023.s001.docx]

S2 Fig. ASMR by location, 2019


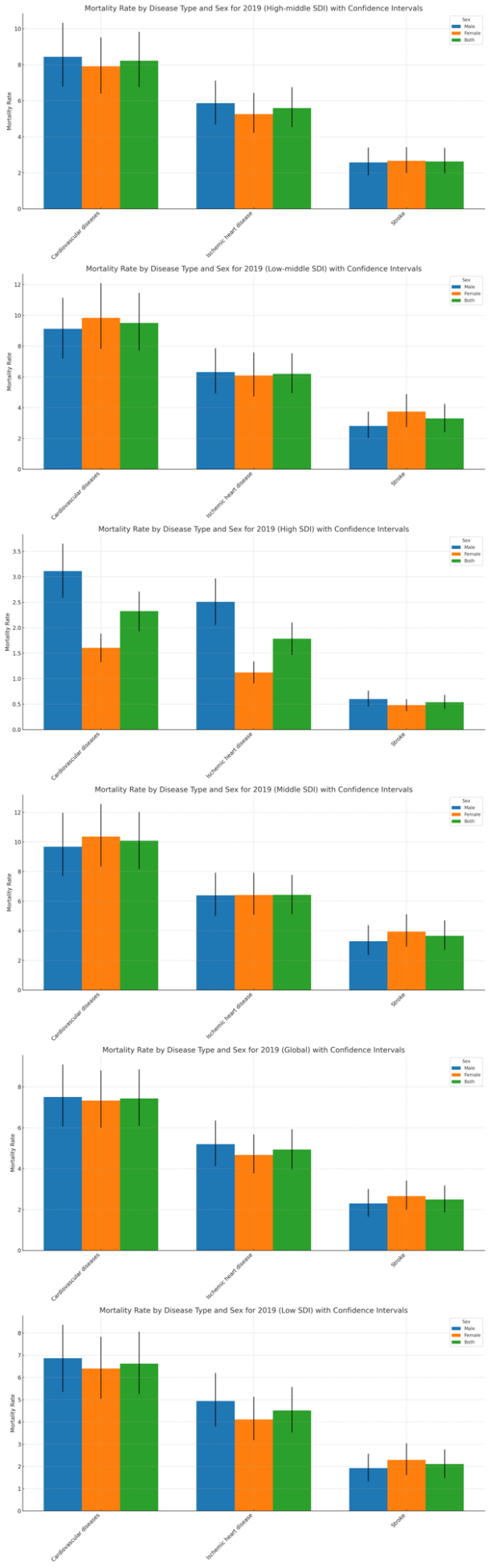

Supplement: S2 Fig — (DOCX) [file pone.0316023.s002.docx]
