# Supplementary material for: Global burden of cardiovascular disease mortality attributable to secondhand smoke, 1990–2019: Systematic analysis of the Global Burden of Disease Study 2019
Source: PLoS One. 2024 Dec 27;19(12):e0316023. doi: 10.1371/journal.pone.0316023 (PMC11676574; doi:10.1371/journal.pone.0316023)
Supplement: S3 Fig — (DOCX) [file pone.0316023.s003.docx]

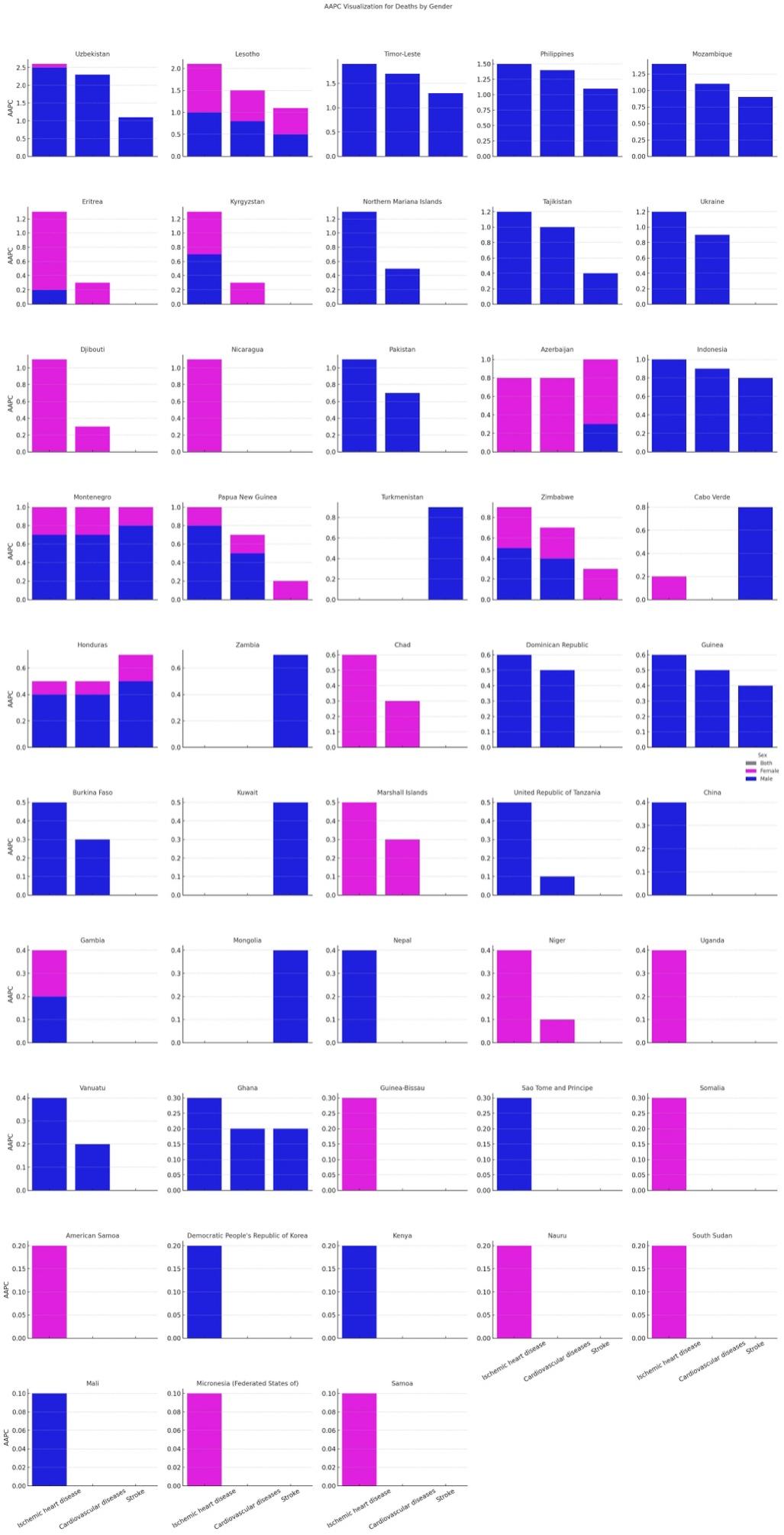
S3 Fig. AAPC of ASMR for cardiovascular diseases attributable to secondhand smoke, stratified by gender and cardiovascular disease type
