# Supplementary material for: Global burden of cardiovascular disease mortality attributable to secondhand smoke, 1990–2019: Systematic analysis of the Global Burden of Disease Study 2019
Source: PLoS One. 2024 Dec 27;19(12):e0316023. doi: 10.1371/journal.pone.0316023 (PMC11676574; doi:10.1371/journal.pone.0316023)

S4 Fig. AAPC of age-standardized DALYs for cardiovascular diseases attributable to secondhand smoke, stratified by gender and cardiovascular disease type


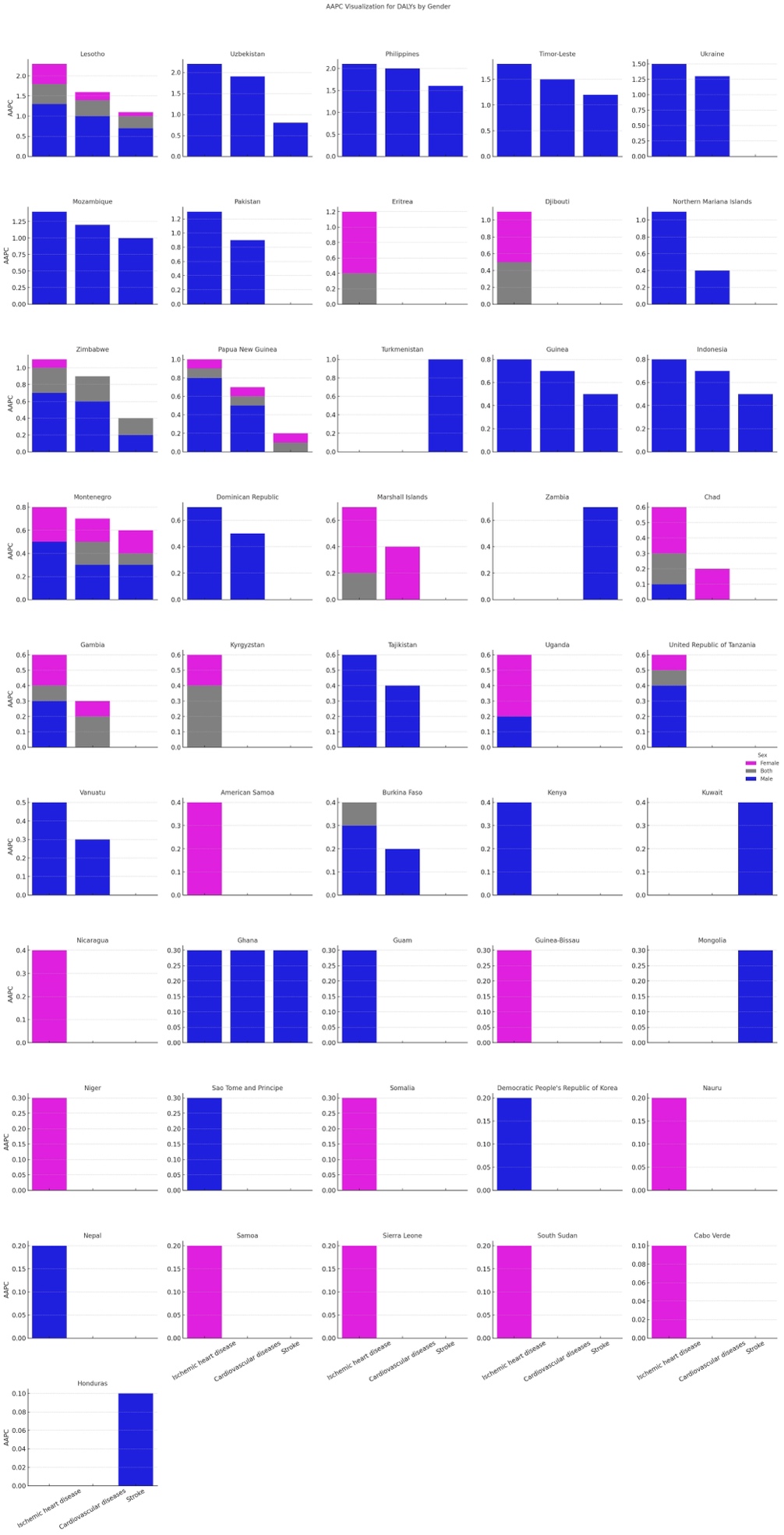

Supplement: S4 Fig — (DOCX) [file pone.0316023.s004.docx]
