# Supplementary material for: Global burden of cardiovascular disease mortality attributable to secondhand smoke, 1990–2019: Systematic analysis of the Global Burden of Disease Study 2019
Source: PLoS One. 2024 Dec 27;19(12):e0316023. doi: 10.1371/journal.pone.0316023 (PMC11676574; doi:10.1371/journal.pone.0316023)
Supplement: S1 Table — (DOCX) [file pone.0316023.s005.docx]

S1 Table. Burden of cardiovascular diseases attributable to secondhand smoke by region, in 2019

| Location | DALYs | ASMR |
| --- | --- | --- |
| Andean Latin America | 38.36(27.2,50.67) | 1.52(1.09,1.99) |
| Australasia | 37.61(30.56,45.28) | 1.51(1.22,1.82) |
| Caribbean | 124.11(95.31,152.89) | 4.95(3.9,6.07) |
| Central Asia | 429.68(350.98,514.83) | 20.88(17.15,24.94) |
| Central Europe | 182.54(144.49,220.44) | 8.94(7.03,10.86) |
| Central Latin America | 77.13(57.12,98.53) | 3.33(2.49,4.26) |
| Central Sub-Saharan Africa | 80.37(57.07,109.43) | 3.12(2.22,4.22) |
| East Asia | 210.29(166.66,259.1) | 10.42(8.23,12.82) |
| Eastern Europe | 289.04(236.92,348.95) | 12.61(10.38,15.03) |
| Eastern Sub-Saharan Africa | 85.02(61.53,110.09) | 3.35(2.44,4.32) |
| High-income Asia Pacific | 37.63(30.76,45.04) | 1.4(1.12,1.66) |
| High-income North America | 66.89(55.22,79.22) | 2.47(2.03,2.91) |
| North Africa and Middle East | 335.97(267.18,402.94) | 14.49(11.62,17.32) |
| Oceania | 476.84(347.01,629.9) | 17.63(12.95,23.04) |
| South Asia | 230.98(181.93,283.81) | 9.32(7.41,11.48) |
| Southeast Asia | 246.08(197,300.08) | 10.26(8.19,12.49) |
| Southern Latin America | 98.48(80.69,117.98) | 4.43(3.63,5.27) |
| Southern Sub-Saharan Africa | 122.92(97.06,148.61) | 5.4(4.25,6.52) |
| Tropical Latin America | 110.41(89.91,132.13) | 4.32(3.51,5.17) |
| Western Europe | 46.94(38.63,55.17) | 1.9(1.57,2.22) |
| Western Sub-Saharan Africa | 100.8(77.7,126.78) | 4.25(3.3,5.3) |
