# Supplementary material for: Global burden of cardiovascular disease mortality attributable to secondhand smoke, 1990–2019: Systematic analysis of the Global Burden of Disease Study 2019
Source: PLoS One. 2024 Dec 27;19(12):e0316023. doi: 10.1371/journal.pone.0316023 (PMC11676574; doi:10.1371/journal.pone.0316023)
Supplement: S2 Table — (DOCX) [file pone.0316023.s006.docx]

S2 Table. Countries with an increased burden of cardiovascular disease attributable to second-hand smoke, by gender

|  | DALYs | | | ASMR | | |
| --- | --- | --- | --- | --- | --- | --- |
| Country | Both | Female | Male | Both | Female | Male |
| Afghanistan | 628.09(455.34,824.79) | 722.57(510.51,969.44) | 535.7(383.87,698.2) | 24.53(18.17,31.4) | 27.33(19.86,35.69) | 21.75(15.74,27.65) |
| Azerbaijan | 550.83(440.08,678.99) | 577.13(447.59,727.31) | 509.26(388.77,657.81) | 29.55(23.57,36.22) | 31.9(24.76,39.84) | 25.77(19.79,33.03) |
| Kiribati | 925.75(677.21,1202.04) | 731.12(524.4,960.67) | 1145.45(838.55,1516.3) | 31.55(23.31,40.7) | 26.4(19.23,34.72) | 37.48(27.89,48.67) |
| Micronesia (Federated States of) | 622.28(402.59,890.13) | 523.72(331.7,763.42) | 716.99(443.71,1045.65) | 22.81(15.85,31.33) | 20.35(14.09,29.24) | 25.02(16.08,35.32) |
| Nauru | 845.48(636.82,1112.59) | 737.77(510.7,1030.87) | 966.63(706.53,1335.82) | 30.15(23.13,38.81) | 27.72(19.91,37.7) | 33.23(24.23,44.66) |
| Solomon Islands | 1183.88(877.01,1562.67) | 1158.16(838.34,1538.49) | 1208.14(881.18,1617.67) | 41.2(31.49,52.79) | 40.8(30.11,52.19) | 41.57(30.99,54.5) |
| Turkmenistan | 666.87(514.2,854.88) | 610.12(471.39,776.94) | 739.29(559.35,963.46) | 30.05(23.26,38.14) | 27.63(21.43,34.78) | 33.48(25.6,43.03) |
| Tuvalu | 605.77(437.1,807.49) | 551.35(390.74,740.87) | 654.53(469.04,893.9) | 22.5(16.51,29.66) | 21.54(15.5,28.55) | 23.21(17.15,31.07) |
| Uzbekistan | 555.44(436.82,690.02) | 517.62(407.14,645.21) | 601.74(463.64,758.22) | 28.89(22.77,35.66) | 27.61(21.84,34.08) | 30.37(23.29,38.39) |
| Yemen | 542.8(401.15,725.81) | 529.27(389.12,723.44) | 557.1(402.18,764.57) | 22.98(17.49,30.28) | 22.2(16.8,29.36) | 23.79(17.21,31.71) |
