# Supplementary material for: Global burden of cardiovascular disease mortality attributable to secondhand smoke, 1990–2019: Systematic analysis of the Global Burden of Disease Study 2019
Source: PLoS One. 2024 Dec 27;19(12):e0316023. doi: 10.1371/journal.pone.0316023 (PMC11676574; doi:10.1371/journal.pone.0316023)
Supplement: S3 Table — (DOCX) [file pone.0316023.s007.docx]

S3 Table. AAPC of age-standardized cardiovascular disease mortality and DALYs attributable to secondhand smoke, stratified by region, 1990-2019

|  |  |  | AAPC | |  |
| --- | --- | --- | --- | --- | --- |
|  |  | Gender | Both | Female | Male |
| Measure | Location | Cause |  |  |  |
| DALYs | Andean Latin America | Cardiovascular diseases | -3.1 (-3.2, -3.0) | -3.4 (-3.6, -3.2) | -2.9 (-3.0, -2.8) |
|  |  | Ischemic heart disease | -2.9 (-3.0, -2.8) | -3.3 (-3.5, -3.2) | -2.8 (-3.0, -2.6) |
|  |  | Stroke | -3.3 (-3.4, -3.3) | -3.5 (-3.7, -3.3) | -3.2 (-3.3, -3.1) |
|  | Australasia | Cardiovascular diseases | -4.8 (-4.8, -4.7) | -4.7 (-4.8, -4.7) | -4.8 (-4.9, -4.8) |
|  |  | Ischemic heart disease | -5.0 (-5.1, -5.0) | -5.2 (-5.3, -5.2) | -4.9 (-5.0, -4.9) |
|  |  | Stroke | -3.8 (-3.8, -3.7) | -3.5 (-3.5, -3.4) | -4.0 (-4.1, -4.0) |
|  | Caribbean | Cardiovascular diseases | -1.9 (-2.0, -1.8) | -2.2 (-2.3, -2.1) | -1.7 (-1.7, -1.6) |
|  |  | Ischemic heart disease | -2.0 (-2.1, -1.9) | -2.4 (-2.4, -2.3) | -1.7 (-1.8, -1.7) |
|  |  | Stroke | -1.7 (-1.8, -1.6) | -1.9 (-2.0, -1.9) | -1.3 (-1.4, -1.3) |
|  | Central Asia | Cardiovascular diseases | -0.2 (-0.3, -0.2) | -0.4 (-0.5, -0.3) | -0.1 (-0.1, 0.0) |
|  |  | Ischemic heart disease | -0.1 (-0.2, -0.1) | -0.2 (-0.3, -0.2) | -0.1 (-0.1, 0.0) |
|  |  | Stroke | -0.6 (-0.6, -0.5) | -0.8 (-0.9, -0.7) | -0.1 (-0.2, -0.1) |
|  | Central Europe | Cardiovascular diseases | -3.0 (-3.0, -2.9) | -3.2 (-3.2, -3.1) | -2.9 (-3.0, -2.8) |
|  |  | Ischemic heart disease | -3.1 (-3.2, -3.0) | -3.3 (-3.3, -3.2) | -3.0 (-3.1, -2.9) |
|  |  | Stroke | -2.7 (-2.8, -2.7) | -3.0 (-3.0, -2.9) | -2.4 (-2.5, -2.4) |
|  | Central Latin America | Cardiovascular diseases | -2.1 (-2.2, -2.1) | -2.7 (-2.7, -2.6) | -1.6 (-1.8, -1.5) |
|  |  | Ischemic heart disease | -2.0 (-2.0, -1.9) | -2.5 (-2.6, -2.4) | -1.5 (-1.6, -1.4) |
|  |  | Stroke | -2.6 (-2.7, -2.6) | -3.1 (-3.1, -3.0) | -2.1 (-2.2, -2.0) |
|  | Central Sub-Saharan Africa | Cardiovascular diseases | -1.1 (-1.2, -1.1) | -0.9 (-0.9, -0.8) | -1.4 (-1.5, -1.4) |
|  |  | Ischemic heart disease | -1.0 (-1.1, -1.0) | -0.6 (-0.6, -0.5) | -1.4 (-1.4, -1.3) |
|  |  | Stroke | -1.3 (-1.3, -1.3) | -1.2 (-1.2, -1.1) | -1.5 (-1.5, -1.5) |
|  | East Asia | Cardiovascular diseases | -1.6 (-1.6, -1.6) | -2.2 (-2.2, -2.1) | -0.8 (-0.9, -0.8) |
|  |  | Ischemic heart disease | -0.6 (-0.6, -0.6) | -1.1 (-1.2, -1.1) | 0.0 (0.0, 0.1) |
|  |  | Stroke | -2.5 (-2.5, -2.4) | -3.0 (-3.1, -3.0) | -1.7 (-1.7, -1.6) |
|  | Eastern Europe | Cardiovascular diseases | -0.6 (-0.8, -0.4) | -1.1 (-1.3, -0.9) | 0.2 (0.0, 0.5) |
|  |  | Ischemic heart disease | -0.4 (-0.6, -0.1) | -0.8 (-1.0, -0.5) | 0.3 (0.0, 0.5) |
|  |  | Stroke | -1.3 (-1.5, -1.1) | -1.8 (-1.9, -1.5) | 0.0 (-0.3, 0.2) |
|  | Eastern Sub-Saharan Africa | Cardiovascular diseases | -1.1 (-1.1, -1.1) | -1.2 (-1.2, -1.2) | -1.0 (-1.0, -1.0) |
|  |  | Ischemic heart disease | -0.7 (-0.7, -0.7) | -0.6 (-0.7, -0.6) | -0.7 (-0.7, -0.7) |
|  |  | Stroke | -1.4 (-1.4, -1.4) | -1.5 (-1.5, -1.5) | -1.3 (-1.3, -1.3) |
|  | High-income Asia Pacific | Cardiovascular diseases | -4.2 (-4.2, -4.1) | -5.0 (-5.1, -5.0) | -3.4 (-3.4, -3.3) |
|  |  | Ischemic heart disease | -4.2 (-4.2, -4.1) | -5.4 (-5.5, -5.4) | -3.3 (-3.4, -3.3) |
|  |  | Stroke | -4.2 (-4.2, -4.1) | -4.7 (-4.8, -4.7) | -3.4 (-3.5, -3.4) |
|  | High-income North America | Cardiovascular diseases | -3.5 (-3.5, -3.4) | -3.1 (-3.1, -3.1) | -3.7 (-3.7, -3.6) |
|  |  | Ischemic heart disease | -3.7 (-3.7, -3.6) | -3.4 (-3.4, -3.3) | -3.8 (-3.9, -3.8) |
|  |  | Stroke | -2.4 (-2.5, -2.4) | -2.4 (-2.4, -2.3) | -2.6 (-2.6, -2.5) |
|  | North Africa and Middle East | Cardiovascular diseases | -1.7 (-1.8, -1.7) | -1.7 (-1.8, -1.7) | -1.8 (-1.8, -1.7) |
|  |  | Ischemic heart disease | -1.8 (-1.8, -1.8) | -1.8 (-1.8, -1.7) | -1.8 (-1.8, -1.8) |
|  |  | Stroke | -1.5 (-1.5, -1.5) | -1.5 (-1.5, -1.5) | -1.5 (-1.5, -1.4) |
|  | Oceania | Cardiovascular diseases | 0.1 (0.1, 0.1) | 0.2 (0.1, 0.2) | 0.1 (0.0, 0.1) |
|  |  | Ischemic heart disease | 0.3 (0.2, 0.3) | 0.4 (0.4, 0.4) | 0.2 (0.2, 0.3) |
|  |  | Stroke | -0.2 (-0.2, -0.2) | -0.1 (-0.2, -0.1) | -0.3 (-0.3, -0.3) |
|  | South Asia | Cardiovascular diseases | -0.7 (-0.7, -0.6) | -0.9 (-1.1, -0.8) | -0.4 (-0.5, -0.3) |
|  |  | Ischemic heart disease | -0.3 (-0.4, -0.2) | -0.6 (-0.8, -0.5) | -0.1 (-0.2, 0.0) |
|  |  | Stroke | -1.3 (-1.4, -1.2) | -1.5 (-1.6, -1.4) | -1.1 (-1.2, -1.1) |
|  | Southeast Asia | Cardiovascular diseases | -0.6 (-0.7, -0.6) | -0.9 (-1.0, -0.9) | -0.1 (-0.2, -0.1) |
|  |  | Ischemic heart disease | -0.4 (-0.4, -0.4) | -0.8 (-0.8, -0.8) | 0.2 (0.1, 0.2) |
|  |  | Stroke | -0.9 (-0.9, -0.8) | -1.1 (-1.1, -1.0) | -0.5 (-0.5, -0.4) |
|  | Southern Latin America | Cardiovascular diseases | -3.4 (-3.4, -3.3) | -3.6 (-3.7, -3.6) | -3.2 (-3.3, -3.2) |
|  |  | Ischemic heart disease | -3.3 (-3.4, -3.2) | -3.6 (-3.7, -3.5) | -3.2 (-3.2, -3.1) |
|  |  | Stroke | -3.5 (-3.6, -3.4) | -3.7 (-3.7, -3.6) | -3.4 (-3.5, -3.3) |
|  | Southern Sub-Saharan Africa | Cardiovascular diseases | -1.5 (-1.8, -1.4) | -1.7 (-1.9, -1.4) | -1.4 (-1.5, -1.3) |
|  |  | Ischemic heart disease | -1.3 (-1.5, -1.2) | -1.2 (-1.5, -1.0) | -1.3 (-1.4, -1.2) |
|  |  | Stroke | -1.9 (-2.1, -1.7) | -2.1 (-2.3, -1.9) | -1.5 (-1.7, -1.5) |
|  | Tropical Latin America | Cardiovascular diseases | -3.8 (-3.9, -3.8) | -3.9 (-4.0, -3.8) | -3.8 (-3.9, -3.7) |
|  |  | Ischemic heart disease | -3.5 (-3.6, -3.5) | -3.6 (-3.6, -3.5) | -3.5 (-3.5, -3.4) |
|  |  | Stroke | -4.4 (-4.5, -4.3) | -4.3 (-4.4, -4.2) | -4.4 (-4.5, -4.3) |
|  | Western Europe | Cardiovascular diseases | -4.2 (-4.2, -4.2) | -4.4 (-4.5, -4.4) | -4.2 (-4.2, -4.1) |
|  |  | Ischemic heart disease | -4.3 (-4.3, -4.2) | -4.6 (-4.7, -4.6) | -4.2 (-4.3, -4.2) |
|  |  | Stroke | -4.0 (-4.0, -3.9) | -4.0 (-4.0, -3.9) | -4.0 (-4.0, -3.9) |
|  | Western Sub-Saharan Africa | Cardiovascular diseases | -0.7 (-0.7, -0.7) | -0.8 (-0.8, -0.7) | -0.7 (-0.7, -0.6) |
|  |  | Ischemic heart disease | -0.5 (-0.5, -0.5) | -0.4 (-0.4, -0.4) | -0.5 (-0.6, -0.5) |
|  |  | Stroke | -1.0 (-1.0, -1.0) | -1.2 (-1.2, -1.1) | -0.8 (-0.9, -0.8) |
| ASMR | Andean Latin America | Cardiovascular diseases | -3.2 (-3.3, -3.0) | -3.3 (-3.4, -3.2) | -3.1 (-3.2, -2.9) |
|  |  | Ischemic heart disease | -2.9 (-3.0, -2.8) | -3.2 (-3.3, -3.0) | -2.8 (-2.9, -2.7) |
|  |  | Stroke | -3.5 (-3.6, -3.4) | -3.5 (-3.7, -3.4) | -3.3 (-3.4, -3.2) |
|  | Australasia | Cardiovascular diseases | -4.8 (-4.9, -4.7) | -4.8 (-4.9, -4.8) | -4.8 (-4.9, -4.8) |
|  |  | Ischemic heart disease | -5.0 (-5.0, -4.9) | -5.2 (-5.2, -5.1) | -4.9 (-5.0, -4.9) |
|  |  | Stroke | -4.0 (-4.1, -3.9) | -3.9 (-4.0, -3.9) | -4.3 (-4.4, -4.2) |
|  | Caribbean | Cardiovascular diseases | -2.0 (-2.0, -1.9) | -2.3 (-2.4, -2.2) | -1.7 (-1.8, -1.6) |
|  |  | Ischemic heart disease | -2.1 (-2.2, -2.0) | -2.5 (-2.6, -2.4) | -1.9 (-1.9, -1.8) |
|  |  | Stroke | -1.6 (-1.7, -1.5) | -1.9 (-2.0, -1.8) | -1.3 (-1.4, -1.2) |
|  | Central Asia | Cardiovascular diseases | 0.0 (0.0, 0.0) | -0.1 (-0.2, -0.1) | 0.2 (0.1, 0.2) |
|  |  | Ischemic heart disease | 0.1 (0.0, 0.1) | 0.0 (-0.1, 0.0) | 0.2 (0.2, 0.3) |
|  |  | Stroke | -0.3 (-0.4, -0.2) | -0.5 (-0.6, -0.5) | 0.1 (0.0, 0.2) |
|  | Central Europe | Cardiovascular diseases | -2.7 (-2.8, -2.7) | -2.9 (-3.0, -2.9) | -2.6 (-2.7, -2.6) |
|  |  | Ischemic heart disease | -2.8 (-2.9, -2.8) | -3.0 (-3.0, -2.9) | -2.8 (-2.8, -2.7) |
|  |  | Stroke | -2.5 (-2.6, -2.5) | -2.8 (-2.8, -2.7) | -2.2 (-2.3, -2.1) |
|  | Central Latin America | Cardiovascular diseases | -2.2 (-2.2, -2.1) | -2.7 (-2.7, -2.6) | -1.7 (-1.8, -1.6) |
|  |  | Ischemic heart disease | -2.0 (-2.1, -1.9) | -2.5 (-2.5, -2.4) | -1.6 (-1.7, -1.5) |
|  |  | Stroke | -2.7 (-2.8, -2.7) | -3.2 (-3.2, -3.1) | -2.3 (-2.4, -2.2) |
|  | Central Sub-Saharan Africa | Cardiovascular diseases | -1.0 (-1.1, -0.9) | -0.8 (-0.9, -0.8) | -1.3 (-1.4, -1.3) |
|  |  | Ischemic heart disease | -0.9 (-0.9, -0.8) | -0.6 (-0.7, -0.6) | -1.2 (-1.3, -1.2) |
|  |  | Stroke | -1.2 (-1.3, -1.2) | -1.1 (-1.1, -1.0) | -1.4 (-1.5, -1.4) |
|  | East Asia | Cardiovascular diseases | -1.3 (-1.3, -1.3) | -1.8 (-1.8, -1.7) | -0.6 (-0.6, -0.6) |
|  |  | Ischemic heart disease | -0.2 (-0.2, -0.1) | -0.6 (-0.7, -0.5) | 0.4 (0.3, 0.4) |
|  |  | Stroke | -2.4 (-2.5, -2.4) | -2.9 (-3.0, -2.9) | -1.6 (-1.6, -1.5) |
|  | Eastern Europe | Cardiovascular diseases | -0.8 (-1.0, -0.6) | -1.2 (-1.4, -1.0) | 0.1 (-0.1, 0.3) |
|  |  | Ischemic heart disease | -0.5 (-0.7, -0.3) | -0.9 (-1.1, -0.7) | 0.1 (-0.2, 0.3) |
|  |  | Stroke | -1.6 (-1.8, -1.4) | -2.0 (-2.2, -1.8) | -0.4 (-0.6, -0.2) |
|  | Eastern Sub-Saharan Africa | Cardiovascular diseases | -0.9 (-1.0, -0.9) | -1.0 (-1.0, -0.9) | -0.9 (-1.0, -0.9) |
|  |  | Ischemic heart disease | -0.6 (-0.7, -0.6) | -0.5 (-0.6, -0.5) | -0.7 (-0.7, -0.7) |
|  |  | Stroke | -1.3 (-1.3, -1.2) | -1.3 (-1.3, -1.3) | -1.2 (-1.2, -1.2) |
|  | High-income Asia Pacific | Cardiovascular diseases | -4.6 (-4.7, -4.6) | -5.5 (-5.6, -5.5) | -3.7 (-3.7, -3.6) |
|  |  | Ischemic heart disease | -4.4 (-4.5, -4.4) | -5.4 (-5.5, -5.3) | -3.5 (-3.5, -3.4) |
|  |  | Stroke | -4.9 (-5.0, -4.9) | -5.6 (-5.7, -5.6) | -3.9 (-4.0, -3.9) |
|  | High-income North America | Cardiovascular diseases | -3.5 (-3.6, -3.5) | -3.3 (-3.4, -3.3) | -3.7 (-3.8, -3.7) |
|  |  | Ischemic heart disease | -3.7 (-3.7, -3.6) | -3.5 (-3.6, -3.5) | -3.9 (-3.9, -3.8) |
|  |  | Stroke | -2.6 (-2.7, -2.6) | -2.5 (-2.6, -2.5) | -2.8 (-2.9, -2.8) |
|  | North Africa and Middle East | Cardiovascular diseases | -1.7 (-1.7, -1.6) | -1.6 (-1.7, -1.6) | -1.7 (-1.7, -1.6) |
|  |  | Ischemic heart disease | -1.7 (-1.7, -1.6) | -1.7 (-1.7, -1.6) | -1.7 (-1.7, -1.7) |
|  |  | Stroke | -1.5 (-1.5, -1.5) | -1.5 (-1.5, -1.5) | -1.5 (-1.5, -1.5) |
|  | Oceania | Cardiovascular diseases | 0.1 (0.1, 0.1) | 0.1 (0.1, 0.2) | 0.1 (0.0, 0.1) |
|  |  | Ischemic heart disease | 0.3 (0.3, 0.3) | 0.3 (0.3, 0.4) | 0.2 (0.2, 0.3) |
|  |  | Stroke | -0.3 (-0.3, -0.3) | -0.2 (-0.2, -0.2) | -0.4 (-0.4, -0.3) |
|  | South Asia | Cardiovascular diseases | -0.8 (-0.9, -0.7) | -1.0 (-1.1, -0.9) | -0.5 (-0.6, -0.4) |
|  |  | Ischemic heart disease | -0.5 (-0.5, -0.4) | -0.7 (-0.8, -0.5) | -0.2 (-0.2, -0.1) |
|  |  | Stroke | -1.5 (-1.6, -1.4) | -1.7 (-1.8, -1.6) | -1.4 (-1.5, -1.2) |
|  | Southeast Asia | Cardiovascular diseases | -0.6 (-0.6, -0.6) | -0.9 (-0.9, -0.8) | -0.1 (-0.2, -0.1) |
|  |  | Ischemic heart disease | -0.4 (-0.4, -0.4) | -0.7 (-0.8, -0.7) | 0.1 (0.1, 0.1) |
|  |  | Stroke | -0.8 (-0.8, -0.8) | -1.0 (-1.0, -1.0) | -0.5 (-0.5, -0.4) |
|  | Southern Latin America | Cardiovascular diseases | -3.4 (-3.5, -3.3) | -3.6 (-3.7, -3.6) | -3.2 (-3.3, -3.1) |
|  |  | Ischemic heart disease | -3.4 (-3.4, -3.3) | -3.7 (-3.8, -3.6) | -3.2 (-3.2, -3.1) |
|  |  | Stroke | -3.4 (-3.5, -3.4) | -3.6 (-3.6, -3.5) | -3.2 (-3.3, -3.2) |
|  | Southern Sub-Saharan Africa | Cardiovascular diseases | -1.3 (-1.5, -1.1) | -1.4 (-1.7, -1.3) | -1.0 (-1.2, -0.9) |
|  |  | Ischemic heart disease | -1.1 (-1.3, -0.9) | -1.2 (-1.4, -1.0) | -1.0 (-1.1, -0.8) |
|  |  | Stroke | -1.6 (-1.7, -1.4) | -1.8 (-2.0, -1.6) | -1.2 (-1.3, -1.1) |
|  | Tropical Latin America | Cardiovascular diseases | -3.9 (-4.0, -3.8) | -3.9 (-4.0, -3.9) | -3.8 (-3.8, -3.7) |
|  |  | Ischemic heart disease | -3.6 (-3.7, -3.5) | -3.7 (-3.8, -3.7) | -3.5 (-3.6, -3.4) |
|  |  | Stroke | -4.3 (-4.4, -4.2) | -4.3 (-4.4, -4.2) | -4.3 (-4.3, -4.2) |
|  | Western Europe | Cardiovascular diseases | -4.2 (-4.3, -4.2) | -4.5 (-4.5, -4.4) | -4.2 (-4.2, -4.1) |
|  |  | Ischemic heart disease | -4.2 (-4.3, -4.2) | -4.5 (-4.6, -4.5) | -4.2 (-4.2, -4.1) |
|  |  | Stroke | -4.2 (-4.2, -4.1) | -4.3 (-4.3, -4.2) | -4.1 (-4.2, -4.1) |
|  | Western Sub-Saharan Africa | Cardiovascular diseases | -0.7 (-0.7, -0.6) | -0.6 (-0.7, -0.6) | -0.6 (-0.7, -0.6) |
|  |  | Ischemic heart disease | -0.4 (-0.4, -0.4) | -0.3 (-0.3, -0.3) | -0.5 (-0.6, -0.5) |
|  |  | Stroke | -0.9 (-1.0, -0.9) | -1.0 (-1.1, -1.0) | -0.8 (-0.8, -0.8) |
