# Supplementary material for: Global burden of cardiovascular disease mortality attributable to secondhand smoke, 1990–2019: Systematic analysis of the Global Burden of Disease Study 2019
Source: PLoS One. 2024 Dec 27;19(12):e0316023. doi: 10.1371/journal.pone.0316023 (PMC11676574; doi:10.1371/journal.pone.0316023)
Supplement: S4 Table — (DOCX) [file pone.0316023.s008.docx]

S4 Table. AAPC of countries with an increased burden of cardiovascular disease deaths attributable to second-hand smoke, by type of cardiovascular disease and gender

| Location | Cause | ASDALYs |  |  | ASMR |  |  |
| --- | --- | --- | --- | --- | --- | --- | --- |
|  |  | Both | Female | Male | Both | Female | Male |
| American Samoa | IHD | Neg. | 0.4 (0.4, 0.5) | Neg. | Neg. | 0.2 (0.1, 0.2) | Neg. |
| Azerbaijan | CVD | Neg. | Neg. | Neg. | 0.5 (0.5, 0.6) | 0.8 (0.7, 0.9) | Neg. |
| Azerbaijan | IHD | Neg. | Neg. | Neg. | 0.5 (0.4, 0.6) | 0.8 (0.7, 0.9) | Neg. |
| Azerbaijan | Stroke | Neg. | Neg. | Neg. | 0.7 (0.6, 0.8) | 1.0 (0.9, 1.1) | 0.3 (0.2, 0.4) |
| BurkiNeg. Faso | CVD | Neg. | Neg. | 0.2 (0.1, 0.3) | 0.2 (0.2, 0.3) | 0.2 (0.1, 0.2) | 0.3 (0.2, 0.4) |
| BurkiNeg. Faso | IHD | 0.4 (0.3, 0.4) | 0.4 (0.3, 0.4) | 0.3 (0.3, 0.4) | 0.4 (0.4, 0.5) | 0.5 (0.5, 0.6) | 0.5 (0.4, 0.5) |
| Cabo Verde | IHD | Neg. | 0.1 (0.1, 0.2) | Neg. | Neg. | 0.2 (0.1, 0.3) | Neg. |
| Cabo Verde | Stroke | Neg. | Neg. | Neg. | Neg. | Neg. | 0.8 (0.4, 1.1) |
| Chad | CVD | Neg. | 0.2 (0.2, 0.3) | Neg. | 0.1 (0.1, 0.2) | 0.3 (0.3, 0.3) | Neg. |
| Chad | IHD | 0.3 (0.3, 0.4) | 0.6 (0.5, 0.6) | 0.1 (0.1, 0.1) | 0.3 (0.3, 0.3) | 0.6 (0.5, 0.6) | Neg. |
| ChiNeg. | IHD | Neg. | Neg. | Neg. | Neg. | Neg. | 0.4 (0.4, 0.5) |
| Democratic People's Republic of Korea | IHD | 0.1 (0.1, 0.1) | Neg. | 0.2 (0.2, 0.2) | 0.1 (0.1, 0.1) | Neg. | 0.2 (0.2, 0.3) |
| Djibouti | CVD | Neg. | Neg. | Neg. | Neg. | 0.3 (0.3, 0.3) | Neg. |
| Djibouti | IHD | 0.5 (0.5, 0.6) | 1.1 (1.0, 1.1) | Neg. | 0.6 (0.6, 0.6) | 1.1 (1.1, 1.2) | Neg. |
| Dominican Republic | CVD | 0.2 (0.1, 0.3) | Neg. | 0.5 (0.3, 0.7) | Neg. | Neg. | 0.5 (0.3, 0.7) |
| Dominican Republic | IHD | 0.4 (0.3, 0.5) | Neg. | 0.7 (0.4, 0.8) | 0.3 (0.1, 0.5) | Neg. | 0.6 (0.4, 0.8) |
| Eritrea | CVD | Neg. | Neg. | Neg. | Neg. | 0.3 (0.3, 0.4) | Neg. |
| Eritrea | IHD | 0.4 (0.4, 0.5) | 1.2 (1.1, 1.2) | Neg. | 0.6 (0.6, 0.7) | 1.3 (1.3, 1.4) | 0.2 (0.1, 0.2) |
| Gambia | CVD | 0.2 (0.1, 0.4) | 0.3 (0.1, 0.4) | Neg. | Neg. | Neg. | Neg. |
| Gambia | IHD | 0.4 (0.3, 0.6) | 0.6 (0.4, 0.8) | 0.3 (0.1, 0.5) | 0.3 (0.2, 0.4) | 0.4 (0.2, 0.5) | 0.2 (0.1, 0.3) |
| GhaNeg. | CVD | Neg. | Neg. | 0.3 (0.2, 0.3) | Neg. | Neg. | 0.2 (0.2, 0.3) |
| GhaNeg. | IHD | Neg. | Neg. | 0.3 (0.2, 0.3) | 0.2 (0.1, 0.2) | 0.1 (0.1, 0.1) | 0.3 (0.2, 0.3) |
| GhaNeg. | Stroke | Neg. | Neg. | 0.3 (0.3, 0.3) | Neg. | Neg. | 0.2 (0.2, 0.3) |
| Guam | IHD | Neg. | Neg. | 0.3 (0.2, 0.4) | Neg. | Neg. | Neg. |
| Guinea | CVD | 0.3 (0.2, 0.3) | Neg. | 0.7 (0.6, 0.7) | 0.2 (0.2, 0.3) | Neg. | 0.5 (0.5, 0.6) |
| Guinea | IHD | 0.6 (0.6, 0.6) | 0.4 (0.4, 0.4) | 0.8 (0.8, 0.9) | 0.5 (0.5, 0.5) | 0.3 (0.3, 0.4) | 0.6 (0.6, 0.7) |
| Guinea | Stroke | Neg. | Neg. | 0.5 (0.4, 0.5) | Neg. | Neg. | 0.4 (0.4, 0.5) |
| Guinea-Bissau | IHD | Neg. | 0.3 (0.3, 0.3) | Neg. | Neg. | 0.3 (0.3, 0.4) | Neg. |
| Honduras | CVD | Neg. | Neg. | Neg. | 0.4 (0.3, 0.5) | 0.5 (0.3, 0.8) | 0.4 (0.4, 0.5) |
| Honduras | IHD | Neg. | Neg. | Neg. | 0.4 (0.3, 0.5) | 0.5 (0.3, 0.8) | 0.4 (0.3, 0.4) |
| Honduras | Stroke | Neg. | Neg. | 0.1 (0.1, 0.2) | 0.5 (0.4, 0.6) | 0.7 (0.5, 0.9) | 0.5 (0.4, 0.5) |
| Indonesia | CVD | Neg. | Neg. | 0.7 (0.7, 0.7) | 0.4 (0.4, 0.4) | 0.1 (0.1, 0.1) | 0.9 (0.9, 0.9) |
| Indonesia | IHD | 0.3 (0.2, 0.3) | Neg. | 0.8 (0.8, 0.8) | 0.5 (0.5, 0.6) | 0.3 (0.2, 0.3) | 1.0 (1.0, 1.0) |
| Indonesia | Stroke | Neg. | Neg. | 0.5 (0.5, 0.5) | 0.2 (0.2, 0.3) | Neg. | 0.8 (0.7, 0.8) |
| Kenya | IHD | 0.2 (0.1, 0.2) | Neg. | 0.4 (0.4, 0.5) | Neg. | Neg. | 0.2 (0.1, 0.2) |
| Kuwait | Stroke | Neg. | Neg. | 0.4 (0.1, 0.8) | Neg. | Neg. | 0.5 (0.2, 0.9) |
| Kyrgyzstan | CVD | Neg. | Neg. | Neg. | 0.3 (0.2, 0.4) | 0.3 (0.2, 0.5) | Neg. |
| Kyrgyzstan | IHD | 0.4 (0.2, 0.5) | 0.6 (0.4, 0.7) | Neg. | 1.1 (0.9, 1.2) | 1.3 (1.1, 1.4) | 0.7 (0.5, 0.9) |
| Lesotho | CVD | 1.4 (1.3, 1.5) | 1.6 (1.5, 1.7) | 1.0 (1.0, 1.1) | 1.3 (1.2, 1.3) | 1.5 (1.5, 1.6) | 0.8 (0.8, 0.8) |
| Lesotho | IHD | 1.8 (1.8, 1.9) | 2.3 (2.2, 2.4) | 1.3 (1.3, 1.4) | 1.6 (1.6, 1.7) | 2.1 (2.0, 2.2) | 1.0 (1.0, 1.1) |
| Lesotho | Stroke | 1.0 (0.9, 1.0) | 1.1 (1.0, 1.2) | 0.7 (0.6, 0.7) | 0.9 (0.9, 1.0) | 1.1 (1.0, 1.2) | 0.5 (0.5, 0.6) |
| Mali | IHD | Neg. | Neg. | Neg. | Neg. | Neg. | 0.1 (0.1, 0.2) |
| Marshall Islands | CVD | Neg. | 0.4 (0.4, 0.4) | Neg. | Neg. | 0.3 (0.3, 0.3) | Neg. |
| Marshall Islands | IHD | 0.2 (0.1, 0.2) | 0.7 (0.6, 0.7) | Neg. | Neg. | 0.5 (0.5, 0.6) | Neg. |
| Micronesia (Federated States of) | IHD | Neg. | Neg. | Neg. | Neg. | 0.1 (0.1, 0.1) | Neg. |
| Mongolia | Stroke | Neg. | Neg. | 0.3 (0.2, 0.5) | Neg. | Neg. | 0.4 (0.2, 0.5) |
| Montenegro | CVD | 0.5 (0.4, 0.5) | 0.7 (0.6, 0.8) | 0.3 (0.2, 0.4) | 0.8 (0.7, 0.9) | 1.0 (0.9, 1.1) | 0.7 (0.6, 0.8) |
| Montenegro | IHD | 0.4 (0.4, 0.5) | 0.8 (0.7, 0.9) | 0.5 (0.3, 0.6) | 0.8 (0.7, 1.0) | 1.0 (0.9, 1.1) | 0.7 (0.5, 0.8) |
| Montenegro | Stroke | 0.4 (0.4, 0.5) | 0.6 (0.6, 0.7) | 0.3 (0.2, 0.4) | 0.8 (0.7, 0.9) | 1.0 (0.9, 1.1) | 0.8 (0.7, 1.0) |
| Mozambique | CVD | 0.6 (0.6, 0.7) | Neg. | 1.2 (1.1, 1.2) | 0.6 (0.6, 0.6) | 0.1 (0.1, 0.1) | 1.1 (1.1, 1.2) |
| Mozambique | IHD | 0.9 (0.9, 1.0) | 0.3 (0.3, 0.4) | 1.4 (1.4, 1.5) | 0.9 (0.9, 0.9) | 0.4 (0.4, 0.4) | 1.4 (1.3, 1.4) |
| Mozambique | Stroke | 0.4 (0.4, 0.4) | Neg. | 1.0 (0.9, 1.0) | 0.4 (0.4, 0.4) | Neg. | 0.9 (0.9, 0.9) |
| Neg.uru | IHD | Neg. | 0.2 (0.2, 0.3) | Neg. | Neg. | 0.2 (0.1, 0.2) | Neg. |
| Nepal | IHD | Neg. | Neg. | 0.2 (0.1, 0.3) | Neg. | Neg. | 0.4 (0.4, 0.5) |
| Nicaragua | IHD | Neg. | 0.4 (0.1, 0.9) | Neg. | 0.6 (0.4, 0.9) | 1.1 (0.7, 1.5) | Neg. |
| Niger | CVD | Neg. | Neg. | Neg. | Neg. | 0.1 (0.1, 0.2) | Neg. |
| Niger | IHD | Neg. | 0.3 (0.2, 0.4) | Neg. | Neg. | 0.4 (0.3, 0.5) | Neg. |
| Northern MariaNeg. Islands | CVD | Neg. | Neg. | 0.4 (0.4, 0.4) | Neg. | Neg. | 0.5 (0.5, 0.6) |
| Northern MariaNeg. Islands | IHD | 0.5 (0.4, 0.5) | Neg. | 1.1 (1.1, 1.1) | 0.5 (0.4, 0.5) | Neg. | 1.3 (1.3, 1.3) |
| Pakistan | CVD | 0.2 (0.1, 0.2) | Neg. | 0.9 (0.9, 0.9) | 0.1 (0.1, 0.1) | Neg. | 0.7 (0.6, 0.7) |
| Pakistan | IHD | 0.5 (0.5, 0.6) | Neg. | 1.3 (1.3, 1.3) | 0.5 (0.4, 0.5) | Neg. | 1.1 (1.1, 1.1) |
| Papua New Guinea | CVD | 0.6 (0.5, 0.6) | 0.7 (0.6, 0.7) | 0.5 (0.5, 0.6) | 0.6 (0.5, 0.6) | 0.7 (0.6, 0.7) | 0.5 (0.5, 0.5) |
| Papua New Guinea | IHD | 0.9 (0.8, 0.9) | 1.0 (0.9, 1.0) | 0.8 (0.8, 0.8) | 0.9 (0.9, 0.9) | 1.0 (0.9, 1.0) | 0.8 (0.8, 0.8) |
| Papua New Guinea | Stroke | 0.1 (0.1, 0.1) | 0.2 (0.2, 0.3) | Neg. | Neg. | 0.2 (0.2, 0.2) | Neg. |
| Philippines | CVD | 1.8 (1.7, 1.9) | 1.6 (1.5, 1.7) | 2.0 (1.9, 2.1) | 1.1 (1.0, 1.2) | 0.8 (0.7, 0.9) | 1.4 (1.3, 1.5) |
| Philippines | IHD | 2.1 (2.0, 2.2) | 2.0 (1.9, 2.1) | 2.1 (2.0, 2.2) | 1.3 (1.2, 1.3) | 1.0 (0.9, 1.1) | 1.5 (1.4, 1.6) |
| Philippines | Stroke | 1.4 (1.3, 1.5) | 1.2 (1.1, 1.3) | 1.6 (1.6, 1.7) | 0.8 (0.7, 0.9) | 0.5 (0.4, 0.6) | 1.1 (1.1, 1.2) |
| Samoa | IHD | Neg. | 0.2 (0.2, 0.2) | Neg. | Neg. | 0.1 (0.1, 0.1) | Neg. |
| Sao Tome and Principe | IHD | 0.3 (0.3, 0.4) | 0.2 (0.1, 0.3) | 0.3 (0.3, 0.4) | 0.3 (0.3, 0.4) | 0.3 (0.2, 0.4) | 0.3 (0.2, 0.3) |
| Sierra Leone | IHD | Neg. | 0.2 (0.1, 0.2) | Neg. | Neg. | Neg. | Neg. |
| Somalia | IHD | Neg. | 0.3 (0.3, 0.3) | Neg. | Neg. | 0.3 (0.2, 0.3) | Neg. |
| South Sudan | IHD | Neg. | 0.2 (0.1, 0.2) | Neg. | Neg. | 0.2 (0.2, 0.2) | Neg. |
| Tajikistan | CVD | Neg. | Neg. | 0.4 (0.3, 0.6) | 0.7 (0.6, 0.8) | 0.4 (0.2, 0.6) | 1.0 (0.9, 1.1) |
| Tajikistan | IHD | 0.5 (0.3, 0.6) | 0.2 (0.1, 0.3) | 0.6 (0.4, 0.7) | 1.0 (0.8, 1.1) | 0.7 (0.5, 1.0) | 1.2 (1.0, 1.4) |
| Tajikistan | Stroke | Neg. | Neg. | Neg. | Neg. | Neg. | 0.4 (0.2, 0.5) |
| Timor-Leste | CVD | 0.4 (0.4, 0.4) | Neg. | 1.5 (1.5, 1.6) | 0.5 (0.5, 0.6) | Neg. | 1.7 (1.6, 1.7) |
| Timor-Leste | IHD | 0.7 (0.6, 0.7) | Neg. | 1.8 (1.7, 1.8) | 0.8 (0.8, 0.9) | 0.1 (0.1, 0.2) | 1.9 (1.9, 2.0) |
| Timor-Leste | Stroke | Neg. | Neg. | 1.2 (1.1, 1.2) | 0.2 (0.2, 0.2) | Neg. | 1.3 (1.3, 1.4) |
| Turkmenistan | Stroke | 0.5 (0.3, 0.8) | Neg. | 1.0 (0.8, 1.3) | 0.4 (0.2, 0.7) | Neg. | 0.9 (0.7, 1.2) |
| Uganda | IHD | 0.2 (0.2, 0.3) | 0.6 (0.5, 0.7) | 0.2 (0.1, 0.2) | Neg. | 0.4 (0.3, 0.4) | Neg. |
| Ukraine | CVD | 0.5 (0.2, 0.8) | Neg. | 1.3 (0.8, 1.7) | Neg. | Neg. | 0.9 (0.6, 1.2) |
| Ukraine | IHD | 0.6 (0.3, 0.9) | Neg. | 1.5 (1.1, 2.0) | 0.3 (0.1, 0.6) | Neg. | 1.2 (0.9, 1.6) |
| United Republic of Tanzania | CVD | Neg. | Neg. | Neg. | Neg. | Neg. | 0.1 (0.1, 0.1) |
| United Republic of Tanzania | IHD | 0.5 (0.4, 0.5) | 0.6 (0.5, 0.6) | 0.4 (0.4, 0.5) | 0.4 (0.3, 0.4) | 0.3 (0.2, 0.3) | 0.5 (0.5, 0.6) |
| Uzbekistan | CVD | 1.7 (1.7, 1.8) | 1.6 (1.6, 1.7) | 1.9 (1.7, 2.0) | 2.2 (2.1, 2.3) | 2.2 (2.1, 2.3) | 2.3 (2.1, 2.4) |
| Uzbekistan | IHD | 2.2 (2.1, 2.3) | 2.2 (2.1, 2.3) | 2.2 (2.0, 2.3) | 2.6 (2.4, 2.7) | 2.6 (2.5, 2.7) | 2.5 (2.4, 2.6) |
| Uzbekistan | Stroke | 0.3 (0.2, 0.4) | Neg. | 0.8 (0.7, 0.9) | 0.8 (0.7, 0.9) | 0.6 (0.5, 0.7) | 1.1 (1.1, 1.2) |
| Vanuatu | CVD | 0.2 (0.2, 0.3) | 0.2 (0.1, 0.3) | 0.3 (0.2, 0.3) | 0.2 (0.1, 0.2) | Neg. | 0.2 (0.2, 0.3) |
| Vanuatu | IHD | 0.4 (0.4, 0.5) | 0.4 (0.3, 0.6) | 0.5 (0.4, 0.5) | 0.4 (0.3, 0.4) | 0.3 (0.2, 0.3) | 0.4 (0.4, 0.5) |
| Zambia | Stroke | Neg. | Neg. | 0.7 (0.6, 0.7) | 0.1 (0.1, 0.2) | Neg. | 0.7 (0.6, 0.7) |
| Zimbabwe | CVD | 0.9 (0.8, 0.9) | 0.9 (0.8, 1.0) | 0.6 (0.5, 0.7) | 0.7 (0.6, 0.7) | 0.7 (0.6, 0.8) | 0.4 (0.3, 0.4) |
| Zimbabwe | IHD | 1.0 (1.0, 1.1) | 1.1 (1.0, 1.2) | 0.7 (0.7, 0.8) | 0.8 (0.8, 0.9) | 0.9 (0.8, 1.0) | 0.5 (0.4, 0.6) |
| Zimbabwe | Stroke | 0.4 (0.4, 0.5) | 0.4 (0.3, 0.5) | 0.2 (0.2, 0.3) | 0.3 (0.2, 0.3) | 0.3 (0.2, 0.4) | Neg. |

ASDALYs = age-standardized DALYs; CVD = Cardiovascular diseases; IHD = Ischemic heart disease; Neg. = negative value
